# Hypertension and Hyperglycemia Synergize to Cause Incipient Renal Tubular Alterations Resulting in Increased NGAL Urinary Excretion in Rats

**DOI:** 10.1371/journal.pone.0105988

**Published:** 2014-08-22

**Authors:** Ana M. Blázquez-Medela, Omar García-Sánchez, Víctor Blanco-Gozalo, Yaremi Quiros, María J. Montero, Carlos Martínez-Salgado, José M. López-Novoa, Francisco J. López-Hernández

**Affiliations:** 1 Instituto de Estudios de Ciencias de la Salud de Castilla y León-Instituto de Investigación Biomédica de Salamanca (IECSCYL-IBSAL), Unidad de Investigación, Hospital Universitario de Salamanca, Salamanca, Spain; 2 Departamento de Fisiología y Farmacología, Universidad de Salamanca, Salamanca, Spain; 3 Instituto Reina Sofía de Investigación Nefrológica, Fundación Iñigo Álvarez de Toledo, Madrid, Spain; 4 Bio-inRen, S.L., Salamanca, Spain; The University of Manchester, United Kingdom

## Abstract

**Background:**

Hypertension and diabetes are the two leading causes of chronic kidney disease (CKD) eventually leading to end stage renal disease (ESRD) and the need of renal replacement therapy. Mortality among CKD and ESRD patients is high, mostly due to cardiovascular events. New early markers of risk are necessary to better anticipate the course of the disease, to detect the renal affection of additive risk factors, and to appropriately handle patients in a pre-emptive and personalized manner.

**Methods:**

Renal function and NGAL urinary excretion was monitored in rats with spontaneous (SHR) or L-NAME induced hypertension rendered hyperglycemic (or not as controls).

**Results:**

Combination of hypertension and hyperglycemia (but not each of these factors independently) causes an increased urinary excretion of neutrophil gelatinase-associated lipocalin (NGAL) in the rat, in the absence of signs of renal damage. Increased NGAL excretion is observed in diabetic animals with two independent models of hypertension. Elevated urinary NGAL results from a specific alteration in its tubular handling, rather than from an increase in its renal expression. In fact, when kidneys of hyperglycaemic-hypertensive rats are perfused *in situ* with Krebs-dextran solution containing exogenous NGAL, they excrete more NGAL in the urine than hypertensive rats. We also show that albuminuria is not capable of detecting the additive effect posed by the coexistence of these two risk factors.

**Conclusions:**

Our results suggest that accumulation of hypertension and hyperglycemia induces an incipient and quite specific alteration in the tubular handling of NGAL resulting in its increased urinary excretion.

## Introduction

Hypertension and diabetes are known to eventually damage target organs including the heart, blood vessels and the kidneys. Hypertension and diabetes often lead to a progressive and irreversible loss of kidney function known as chronic kidney disease (CKD). Hypertensive and diabetic CKD take place during months or years, finally leading to end-stage renal disease (ESRD) [1], [2], characterized by renal failure and the absolute need of renal replacement therapy (RRT), in the form of renal transplant or dialysis [1], [3]. Diabetes and hypertension are the world leading causes of ESRD [4]. The presence of an increasing number of comorbid conditions increases in turn the risk of cardiovascular episodes or of inception and progression of chronic diseases. However, the additive effect of comorbidities on risk statement is hitherto bereft of quantitative markers, which might be useful for stratifying patients pre-emptively according to their individual risk.

Controlling hypertension and hyperglycemia is the most effective strategy to minimize hypertensive and diabetic nephropathies. However, effective treatments are missing for halting CKD progression and reversing the accumulated injury. The most effective drugs available (most prominently, inhibitors of the renin-angiotensin system) are only capable of slowing down renal disease progression [5], [6]. Still, they are useful for preventing the need of RRT during the patient’s lifetime, in some cases; or for postponing RRT installation and thus reducing the time during which it is applied. This has an enormous health and socioeconomic repercussion, because of the impairment in life quality and the high and disproportionate cost derived from dialysis, which consumes about 2% of the total health expenditure, whereas it is applied to 0.1% of the population [7]–[10]. Consequently, a critical aspect of CKD handling, both from the sanitary and the economic points of view, is the effective and early diagnosis. Traditionally, CKD diagnosis has been mostly performed through the detection of signs of renal dysfunction, most prominently of glomerular filtration estimated from the plasma creatinine concentration. However, the equivalent of over 60% of the renal mass is voided by the time renal dysfunction symptoms appear. For this reason, the discovery of earlier markers poses an urgent challenge in order to enhance therapeutic efficacy. The most developed of these new markers is the urinary excretion of albumin. Over excretion of small amounts of albumin (30–300 mg/day; termed microalbuminuria) is detected early in the course of diabetic and hypertensive nephropathy before other markers are evident [11]. Microalbuminuria is the result of subtle alterations in the sieving properties of the glomerular filtration barrier, in tubular reabsorption, or both. Accumulated clinical information has demonstrated that early microalbuminuria is a factor of progression and bad prognosis [11]. The level of circulating tumor necrosis factor (TNF) receptors has also been shown to predict renal function decline [12], [13] and ESRD [13]–[15] in diabetic patients.

Another promising marker under development is urinary neutrophil gelatinase-associated lipocalin (NGAL). NGAL is normally found in the blood. However, its serum and urinary levels increase during inflammation, renal and cardiovascular diseases, certain types of cancer and other conditions [16]. Because of this, this molecule has gained attention in the last decade as a potential biomarker [17]. Indeed, urinary NGAL (uNGAL) has been developed most intensively as an early and sensitive marker of acute kidney injury [18]. It anticipates the course of disease earlier than conventional markers, such as serum creatinine, and has an enhanced prognostic value [19]. Urinary NGAL also correlates with the renal damage inflicted by cisplatin [20], [21], cyclosporine [22] and with outcome after kidney allograft transplantation [23]; and it has been suggested as a potential marker of the transition from AKI to CKD [24]. Moreover, serum and urinary NGAL have been shown to correlate with different chronic renal diseases of glomerular and tubular origin [16], [25], [26], including diabetic nephropathy in mice [27] and humans [28], and hypertensive nephropathy [29]. In this study, we attempted to study the utility of the evolution of uNGAL to detect and monitor potential additive effects of the comorbid factors hypertension and diabetes in the rat.

## Methods

All reagents were purchased from Sigma (Madrid, Spain), except where otherwise indicated.

### Animals and experimental protocol

Male Wistar (Animal Experimentation Service of the University of Salamanca, Salamanca, Spain) and SHR rats (Charles River, Barcelona, Spain) weighing 200–250 g were used. Rats were treated in accordance with the Declaration of Helsinki Principles on the Advice on Care and Use of Animals referred to in: law 14\/2 007 (3 July) on Biomedical Research, Conseil de l’Europe (published in Official Daily N. L358/1-358/6, 18-12-1986), Government Spanish (Royal Decree 223/1 988, (14 March) and Order of 13-10-1989, and Official Bulletin of the State b. 256, pp. 31349–31362, 28-10-1990). The procedure was approved by the Bioethics Committee of the University of Salamanca. Animals were treated with care through the experiments. Except when allocated in metabolic cages for short periods of time (2–3 days), rats were housed 6 per cage under controlled environmental conditions with regulated light/darkness cycles in the University of Salamanca Experimental Animal Service. Rats were fed ad libitum with regular chow and they had free access to water. All surgical procedures were performed under inhalational anaesthesia with 3% isoflurane (2-chloro-2-(difluoromethoxy)-1,1,1-trifluoro-ethane; Schering Plough, Madrid), in 1 L/min oxygen flow. A single dose of buprenorphine (0.01 mg/kg) was administered before awakening to minimize post-surgical pain. Humane policy was implemented, although it was rarely necessary in our experiments. Rats showing evident behavioural signs of illness, pain or distress at any time, including reduced movement, abnormal resting postures, voluntary starvation, ruffled hair, etc. were immediately euthanized with CO_2_.

Rats were rendered diabetic (or not, as controls) with a single injection of streptozotocin (60 mg/kg body weight), and monitored during 3 months. A subset of control and diabetic Wistar rats was treated with L-nitro-arginine methyl ester (L-NAME; 40 mg/kg/day in the drinking water for 7 weeks), starting one week after streptozotocin injection, as a second model of hypertension (combined or not with diabetes). Blood pressure was monitored in conscious animals by the tail cuff method (Cibertec, Madrid, Spain). For glycemic control, diabetic rats were injected daily with the necessary dose of insulin to keep glycemia at about 400 mg/dL. Glycemia was measured weekly with commercial reactive strips (Bayer, Leverkusen, Germany) in a drop of blood from the tail. At different time points, rats were allocated in individual metabolic cages for 24-hour urine sample collection. Urine was cleared by centrifugation, and it was stored at −80°C until use. At different time points after streptozotocin injection, rats were anaesthetized, and the kidneys were perfused by the aorta with heparinised saline solution (0.9% NaCl) and immediately dissected. Animals were killed by exsanguination under anaesthesia. One kidney was frozen in liquid nitrogen and subsequently kept at −80°C for Western blot studies. The other one was fixed in buffered 3.7% p-formaldehyde for histological studies. Blood samples were also obtained in heparinized capillaries at different time points by a small incision in the tail tip. Blood was centrifuged and serum was kept at −80°C until use.

### Histological studies

Paraffin blocks were made with fixed kidneys and 5-µm tissue sections were stained with Masson’s trichrome for the evaluation of fibrosis. Photographs were taken under an Olympus BX51 microscope connected to an Olympus DP70 colour, digital camera (Olympus, Tokyo, Japan).

### Biochemical measurements

Serum and urinary creatinine (Cr_s_ and Cr_u_ respectively) and blood urea concentration were measured by means of the automated analyzer Reflotron (Roche Diagnostics, Barcelona, Spain; lower detection limit of 0.5 mg/dL). Urine protein concentration was measured by the Bradford method [30]. Urine NAG content was determined by a colorimetric method with a commercial kit (Roche Diagnostics, Barcelona, Spain) based on the conversion of 3-cresolsulfonphthaleinyl-N-acetyl-β-D-glucosaminide into the purple 3-cresol-cresolsulfonphthaleinyl. Albuminuria was measured with a commercial ELISA following the manufacturer’s instructions (Bethyl Laboratories, Montgomery, TX, USA).

### Western blot

Western blots were run with (i) urine samples (a volume of urine from each animal corresponding to the same excretion fraction, up to 24 µL of urine; or 21 µL per sample in kidney perfusion experiments), (ii) tissue extracts (100 µg total protein per sample) prepared by homogenizing the kidneys with a tissue mixer (Ultra-Turrax T8, IKA-Werwe) at 4°C in homogenization buffer (140 mM NaCl, 20 mM Tris-HCl pH = 7.5, 0.5 M ethylenediaminetetraacetic acid –EDTA-, 10% glycerol, 1% Igepal CA-630, 1 µg/mL aprotinin, 1 µg/mL leupeptin, 1 µg/mL pepstatin A, 1 mM phenylmethylsulphonyl fluoride –PMSF-), or (iii) albumin-free blood serum. Albumin was removed from serum with a column-based, commercial kit based on the immunological retention of rat albumin (Qproteome Murine Albumin Depletion Kit, Quiagen). Samples were separated by electrophoresis in 10–15% acrylamide gels (Mini Protean II system, BioRad, Madrid, Spain). Immediately, proteins were electrically transferred to an Immobilon-P membrane (Millipore, Madrid, Spain). Membranes were probed with antibodies against NGAL (MBL, Woburn, MA, USA) and albumin (Abcam, Cambridge, UK). An anti-GM2AP polyclonal antibody was produced and used as described [31]. A control peptide for NGAL (MBL, Woburn, MA, USA) was also used in some experiments to ascertain the specificity of the NGAL signal, according to the manufacturer’s instructions.

#### Gene expression analysis

RT-PCR-amplification of NGAL and GAPDH was performed on mRNA samples obtained from kidney tissue with the next primers: for rat NGAL, 5′-TCTGGGCCTAAGGATAACAAC-3′ and 5′-AGACAGGTGGGACCTGAACCA-3′; for rat GAPDH, 5′-TGAAGGTCGGTGTGAACGGATTTGGC-3′ and 5′-CATGTAGGCCATGAGGTCCACCAC-3′. PCR conditions were: 1×(95°C×5 min); n×(95°C×1 min+T_m_×1 min+72°C×1 min); 1×(72°C×5 min); where n was 35 for NGAL and 30 for GAPDH, and T_m_ was 64°C for NGAL, and 55.9°C for GAPDH.

#### Excretion studies with *in situ* perfused kidneys

At the end of the treatment, some hyperglycaemic SHR rats were anesthetized and an extracorporeal circuit for kidney perfusion was set up, as described elsewhere [32], with some modifications. Briefly, the renal artery, vein and ureter of the right kidney were ligated. The renal artery and vein of the left kidney and the urinary bladder were cannulated. Oxygenated and warm (37°C) Krebs-dextran [40 g/L of dextran (molecular weight 64 K–76 K) in Krebs solution (118.3 mM NaCl, 4.7 mM KCl, 1.8 mM CaCl_2_, 1.2 mM MgSO_4_, 1.2 mM KH_2_PO_4_, 25 mM NaHCO_3_, 0.026 mM EDTA, 11.1 glucose, pH = 7.4)] was perfused through the renal artery at 3 mL/min, and was discarded through the renal vein. Urine fractions were collected from a catheter placed in the urinary bladder, starting before the perfusion with Krebs (when blood was still passing through the kidney), and during 2 hours after perfusion with Krebs started. In addition, similar experiments were carried out in hyperglycaemic and normoglycemic SHR, in which an excess of rat NGAL (42 ng/mL; Adipogen, San Diego, CA, USA) was added to the Krebs-dextran solution. As a control of these perfusion experiments, in order to discard a potential artefact derived from surgery or other experimental manoeuvres that would impede NGAL urinary excretion, kidneys were also perfused *in situ* with blood from the carotid artery. For this purpose, the artery, vein and ureter of the left kidney were ligated as above. A catheter was placed in the right carotid artery and connected directly to the renal artery. Urine was collected as above.

In a separate set of experiments, kidney perfusion experiments were carried in normoglycemic Wistar rats. Urine samples were collected from the urinary bladder before and after a bolus injection of sodium maleate (400 mg/kg) or saline (as control) was administered through a catheter placed into the right jugular vein. Sodium maleate was used to block megalin-mediated proximal tubule reabsorption [33], [34]. All urine samples were kept at −80°C until further assayed.

### Statistical analysis

Data are represented as the mean ± standard error of n experiments performed, as indicated in each case. Statistical comparisons were assessed by the one-way ANOVA analysis followed by the post hoc Tukey’s test for multi-group comparisons, and the Student’s t test for comparison between two groups. A p<0.05 was considered statistically significant.

## Results

### Evolution of blood pressure, glycemia and renal function in SHR and Wistar, normo and hyperglycemic rats

**Figure 1:**
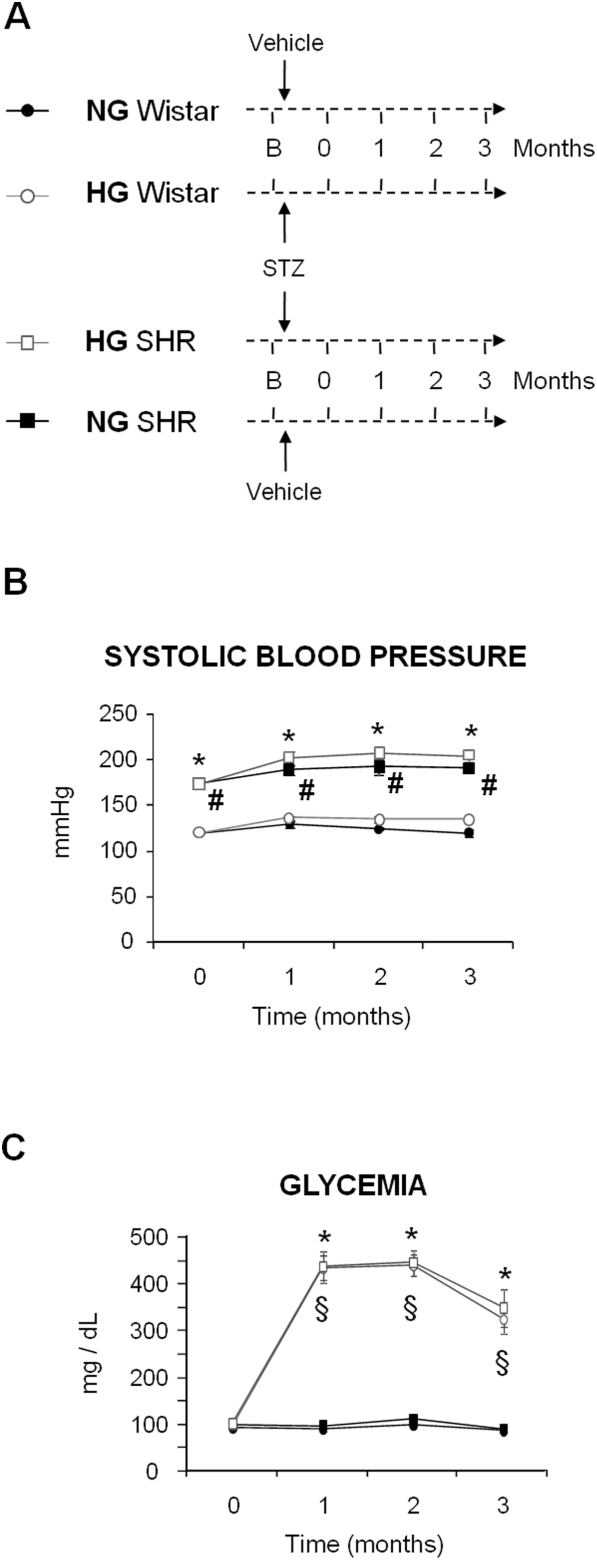
Experimental model, blood pressure and glycemia. Evolution of systolic blood pressure (B; n = 10–12 per group), glycemia (C; n = 10–12 per group), during 3 months in normoglycemic (NG) and hyperglycemic (HG) Wistar and SHR rats. Panel A shows a schematic representation of the experimental model. Data represent the mean ± standard error. *p<0.01 vs. NG Wistar. #p<0.01 vs. HG Wistar. §p<0.01 vs. NG SHR.

**Figure 2:**
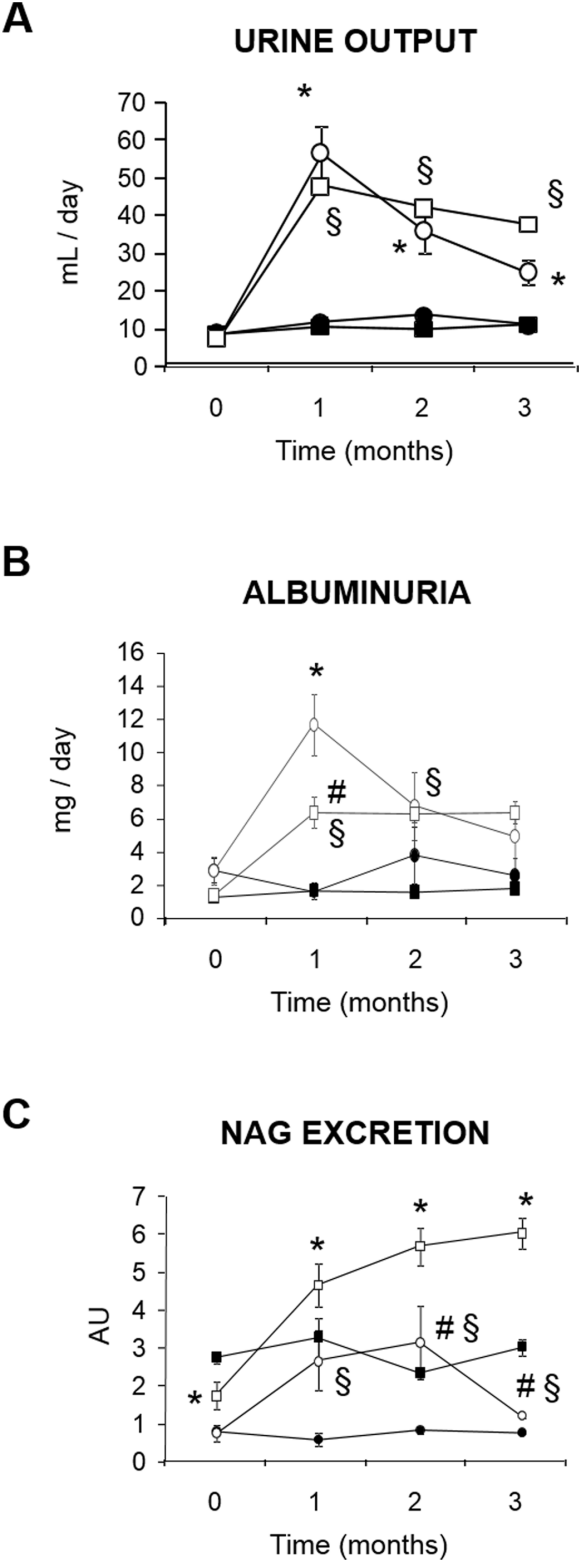
Urinary biochemistry of renal function. Urine output (A; n = 10–12 per group), albuminuria (B; n = 4–6 per group), and NAG excretion (C; n = 4–5 per group) during 3 months in normoglycemic (NG) and hyperglycemic (HG) Wistar and SHR rats. Data represent the mean ± standard error. *p<0.01 vs. NG Wistar. #p<0.01 vs. HG Wistar. §p<0.01 vs. NG SHR.

**Figure 3:**
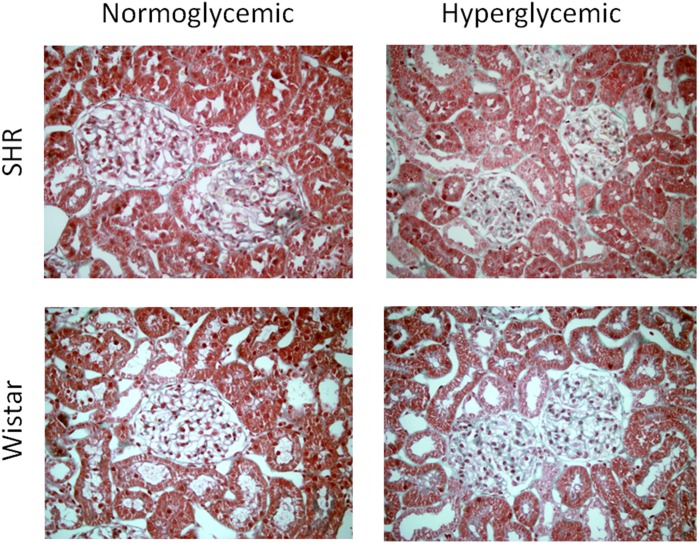
Histological study. Representative images of renal tissue sections stained with Masson’s trichrome 3 months after the inception of hyperglycemia (or not, as control) in Wistar and SHR rats. n = 4 animals per group.

Blood pressure was significantly higher in SHR than in Wistar through the study. However, no differences in BP were seen between normoglycemic and hyperglycemic rats, either from the SHR or the Wistar strain (figure 1-B). Hyperglycemia was induced in a subset of SHR and Wistar by an injection of streptozotocin (STZ). Glycemia was higher in STZ-treated rats than in controls of either strain, and hyperglycemia was very similar in SHR and Wistar rats (figure 1-C). No renal dysfunction was observed in any of the experimental groups through the study, as evidenced by similar values of plasma creatinine, plasma urea, proteinuria (table 1). Microalbuminuria (figure 2) and N-acetylglucosaminidase (NAG) excretion (figure 2) were higher in hyperglycemic SHR and Wistar rats, compared with their corresponding normoglycemic controls. Microalbuminuria and NAG excretion partly correlated with the profile of urinary output (figure 2), which suggests that a part of their excretion might be due to a wash-out effect rather than to renal injury, as described for other proteins [35]. In any case, no further tubular alteration (if any) was induced by the concomitant presence of hypertension and hyperglycemia. Furthermore, microalbuminuria, NAG and sodium excretion did not correlate with the coexistence of these two risk factors, but merely (if at all) with direct or indirect tubular alterations, adaptations or effects caused exclusively by hyperglycemia. No renal tissue injury was evident on the histological study of renal tissue sections (figure 3). Masson’s trichrome staining reveals no signs of fibrosis in any of the experimental conditions, compared to the normal kidney (i.e. in the control group). Furthermore, no gross structural alterations are induced by hyperglycemia, hypertension or the combination of both at this experimental time. Renal corpuscles and tubuli show normal appearance.

**Table 1:** Evolution of plasma creatinine and urea concentration, and urinary protein excretion during 3 months in normoglycemic (NG) and hyperglycemic (HG) Wistar and SHR rats.

Group	Time (months)	Plasma Creatinine (mg/dL)	Plasma Urea (mg/dL)	Urinary Protein Excretion (mg/day)
**NG Wistar**	0	<0.5	N.D.	10.4±2.9
	1	<0.5	N.D.	12.9±2.4
	2	<0.5	N.D.	20.1±1.8
	3	<0.5	N.D.	17.9±2.8
**HG Wistar**	0	<0.5	N.D.	9.5±1.7
	1	<0.5	N.D.	28.2±3.5
	2	<0.5	N.D.	31.2±4.0
	3	<0.5	N.D.	26.5±4.5
**NG SHR**	0	<0.5	42.8±3.9	25.1±2.1
	1	<0.5	45.4±2.5	20.3±2.6
	2	<0.5	40.4±2.0	15.6±1.9
	3	<0.5	41.1±2.2	14.7±1.5
**HG SHR**	0	<0.5	37.4±3.5	25.0±1.4
	1	<0.5	62.8±5.5	17.9±4.3
	2	<0.5	54.9±7.2	38.7±3.0
	3	<0.5	49.9±3.2	35.9±4.9

Data represent the mean ± standard error of n = 6 animals per condition. N.D., not determined.

### NGAL urinary excretion is increased in spontaneously hypertensive-diabetic rats

**Figure 4:**
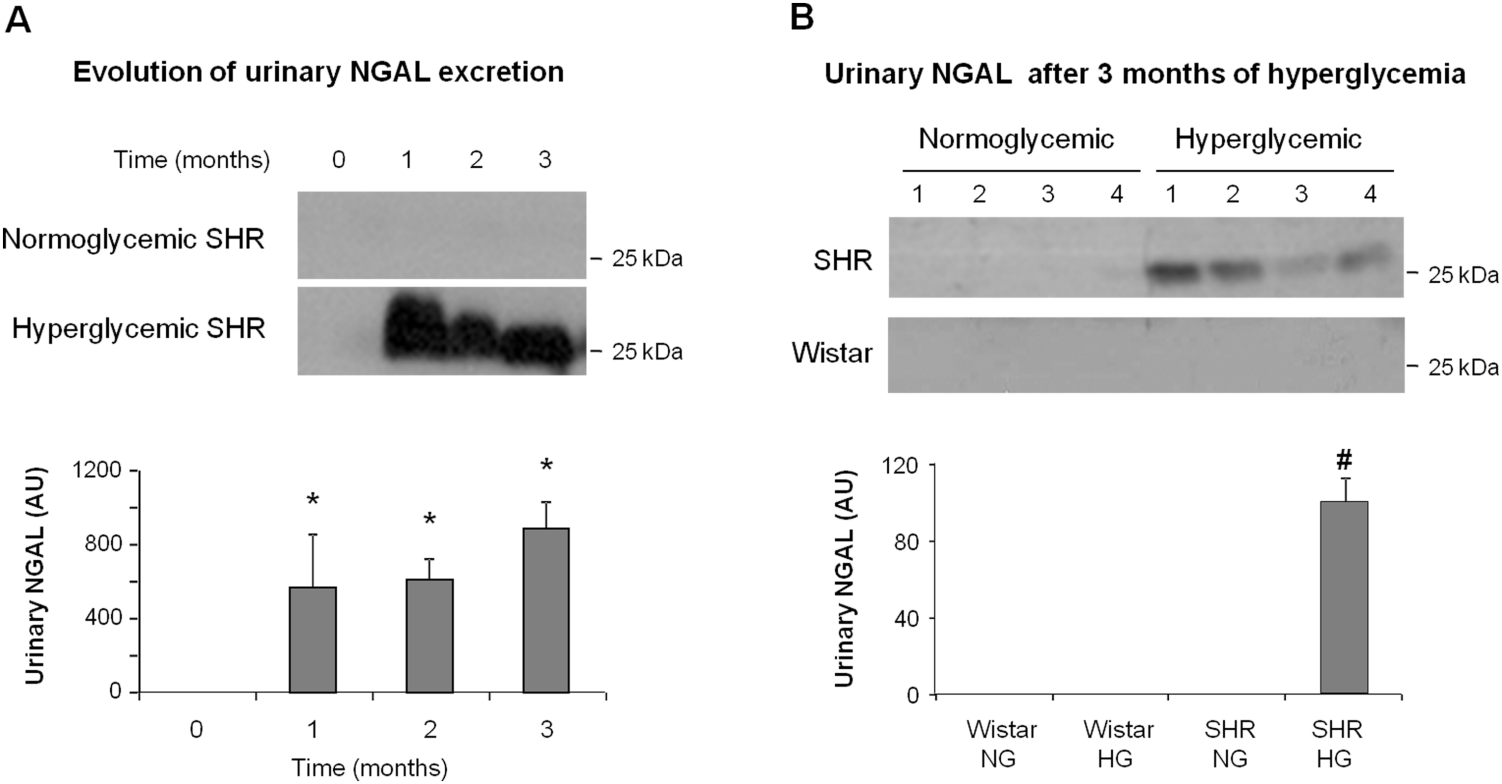
Effect of hyperglycemia and genetic hypertension on NGAL urinary levels. Evolution of NGAL urinary excretion during 3 months (A; n = 3 per group), and level of urinary NGAL after 3 months in different animals per group (B; n = 4 per group), determined by western blot in the urine of normoglycemic (NG) and hyperglycemic (HG) Wistar and SHR rats. Data represent the mean ± standard error. *p<0.01 vs. time 0. #p<0.01 vs. NG Wistar, HG Wistar and NG SHR.

Urinary NGAL excretion, a marker associated to a number of pathological circumstances including early diabetic nephropathy, was not modified by either hypertension or hyperglycemia alone in our experimental setting. However, it increased significantly in rats suffering concomitantly of hypertension (SHR rats) and hyperglycemia (figure 4). Urinary NGAL excretion increased from the first month of hyperglycemia in SHR, and stayed high during the rest of the study.

### NGAL urinary excretion is also increased L-NAME-induced hypertensive-diabetic rats

**Figure 5:**
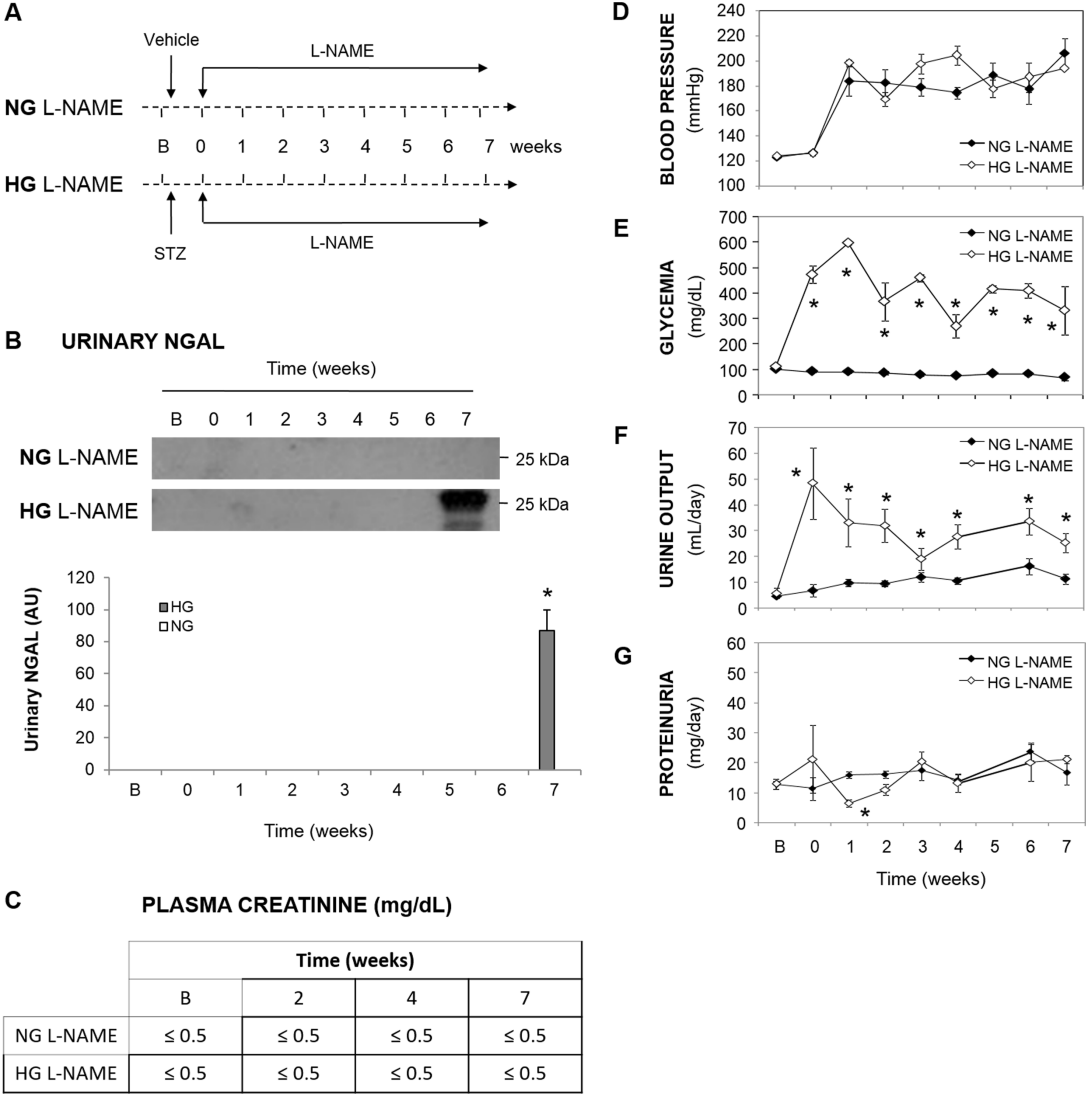
Effect of hyperglycemia and L-NAME-induced hypertension on NGAL urinary levels. Evolution of NGAL urinary excretion and its corresponding densitometric quantification (B; n = 4 per group), plasma creatinine concentration (C; n = 4–6 per group), systolic blood pressure (D; n = 8 per group), glycemia (E; n = 8 per group), urine output (F; n = 8 per group) and proteinuria (G; n = 8 per group) during 7 weeks in Wistar rats rendered hypertensive with L-NAME, in which hyperglycemia was induced in parallel (or not, as control). Panel A shows a schematic representation of the experimental model. Data represent the mean ± standard error. *p<0.001 vs. NG L-NAME. B, basal time point. HG, hyperglycemic. NG, normoglycemic.

In order to verify that the increased urinary excretion of NGAL was the consequence of the combined effect of hypertension and hyperglycemia, and not of a particular characteristic of the SHR strain when rendered hyperglycaemic, we also studied the effect of hyperglycemia on the excretion of this marker in a model of induced hypertension. For this purpose, we induced hypertension in Wistar rats by chronic treatment with the NO synthase inhibitor L-NAME. As shown in figure 5-D, L-NAME-treated rats became rapidly hypertensive. Hyperglycemia was also induced in a subset of animals, which was maintained at values of blood glucose concentration similar to those in SHR (figure 5-E). Renal function was not impaired in L-NAME-hypertensive and L-NAME-hypertensive-hyperglycemic rats, as indicated by the evolution of plasma creatinine concentration and proteinuria (figure 5-C and 5-G, respectively). Urine output increased in hyperglycemic rats (figure 5-F), probably as a consequence of hyperglycemia. In this setting, NGAL was not increased in the urine in the L-NAME-induced hypertension model. However, when L-NAME treated rats were also rendered hyperglycemic, NGAL appeared in the urine after 7 weeks of hypertension (figure 5-B). This indicates that chronic coexistence of both factors is necessary to induce a synergistic increase in NGAL urinary excretion.

### Increased urinary NGAL results from its altered tubular handling

**Figure 6:**
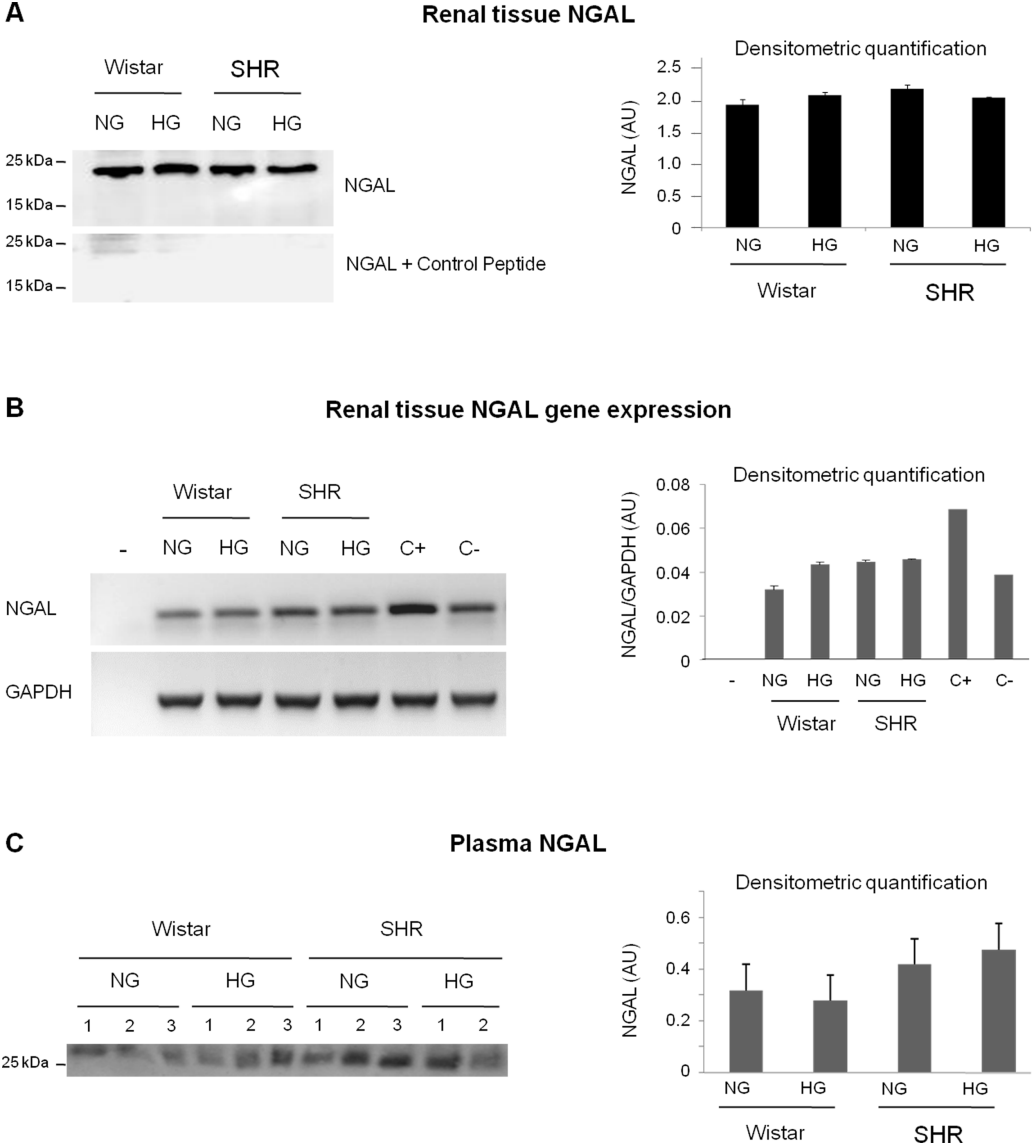
Renal and plasma NGAL are not modified by hyperglycemia and hypertension. Renal tissue NGAL protein levels (A; n = 4–5 per group), renal NGAL gene expression analysis (B; n = 3) and NGAL plasma level (C; n = 2–3 per group), in normoglycemic (NG) and hyperglycemic (HG) Wistar and SHR rats after 3 months of hyperglycemia. In each panel, representative images of Western blot are shown (left) along with the corresponding densitometric quantification (right). In panel B, the next symbols were used: −, H_2_O was added in lieu of template DNA (as a PCR control); C+, a renal tissue sample from a ischemia/reperfusion-injured Wistar rat kidney (as a positive control of increased NAGL expression); C−, a renal tissue sample from a sham-operated Wistar rat (as a control for C+).

**Figure 7:**
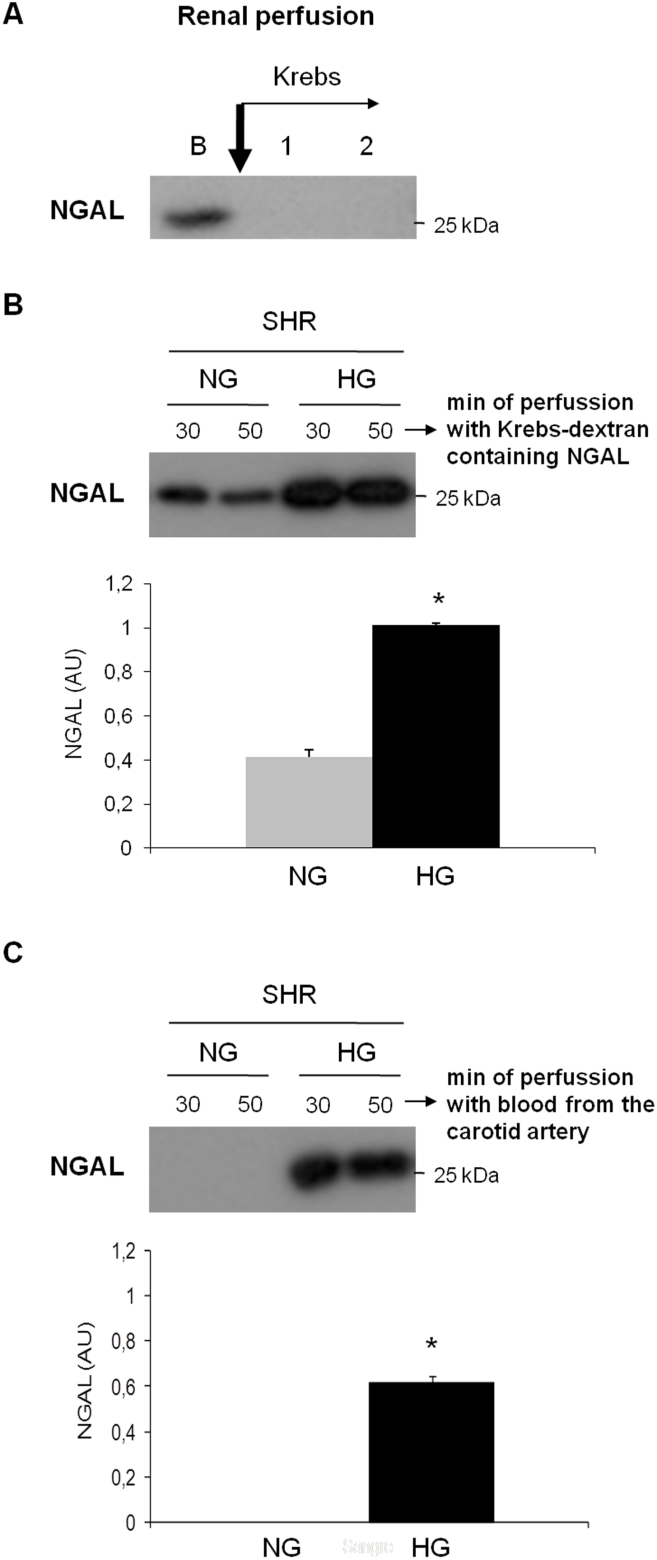
Increased NGAL urinary excretion is due to a tubular handling alteration. A) Representative image of Western blot showing the effect of in situ perfusion of the renal vasculature with Krebs-dextran solution on NGAL urinary excretion in 3-month hyperglycemic SHR rats. B represents a basal urine sample before perfusing Krebs solution, when blood still perfuses the kidney. B) Representative image of Western blot showing the effect of adding an excess of NGAL to the Krebs-dextran solution on NGAL urinary excretion in 3-month normoglycemic (NG) and hyperglycemic SHR; and band quantification (lower graphic). C) Representative image of Western blot showing the effect of renal perfusion with the animal’s own blood from the carotid artery on NGAL urinary excretion in 3-month normoglycemic (NG) and hyperglycemic SHR; and band quantification (lower graphic). Data represent the average ± standard error of n = 5 per group. *p<0.001 vs. normoglycemic (NG) SHR. AU, arbitrary units.

We also aimed at unraveling the origin of the increased urinary NGAL. Figure 6-A shows that the renal tissue NGAL level is not increased in NGAL-excreting rats, that is, in hypertensive-hyperglycemic rats. The reactive band appears a little lower than the typical 25 kDa band found in the urine by us and other authors [36], as shown in the positive control (C+). This could mean that the observed band might not correspond to NGAL. In any case, no increased expression of NGAL in the renal tissue seems to be able to explain the increased urinary excretion. Moreover, gene expression analysis by RT-PCR showed that neither hypertension or diabetes, nor the combination of both modified the expression of NGAL in renal tissue (figure 6-B). As such, the only other possible source of the urinary NGAL is the blood irrigating the kidneys, which is partially filtered through the glomerular filtration barrier. In fact, when the kidney of hypertensive-hyperglycemic rats is perfused with Krebs solution (which does not contain proteins, but a high molecular weight dextran to compensate for the oncotic pressure of the blood), NGAL is not excreted with the urine any more (figure 7-A). This supports the idea that the NGAL observed in the urine of these rats comes from the blood and not from the renal tissue. Moreover, when exogenous rat NGAL was added to the Krebs solution perfusing the kidney, hypertensive-hyperglycemic rats excreted more NGAL in the urine than hypertensive (normoglycemic) rats (figure 7-B). This further indicates that the renal handling of NGAL is intrinsically altered in hypertensive-hyperglycemic rats. As a control of the perfusion experiments, in order to discard an experimental artifact due to the surgical procedure, when the kidneys of hypertensive-hyperglycemic rats were perfused with their own blood through a catheter connecting the carotid artery with the renal artery, NGAL still appeared in the urine (figure 7-C); whereas no NGAL was detected in these circumstances in the urine of hypertensive (normoglycemic) rats. Of note, this latter difference in NGAL excretion when kidneys are perfused in situ with Krebs-dextran containing NGAL is almost identical to the difference in NGAL excretion between hypertensive-hyperglycemic and hypertensive rats when blood perfuses the kidneys.

**Figure 8:**
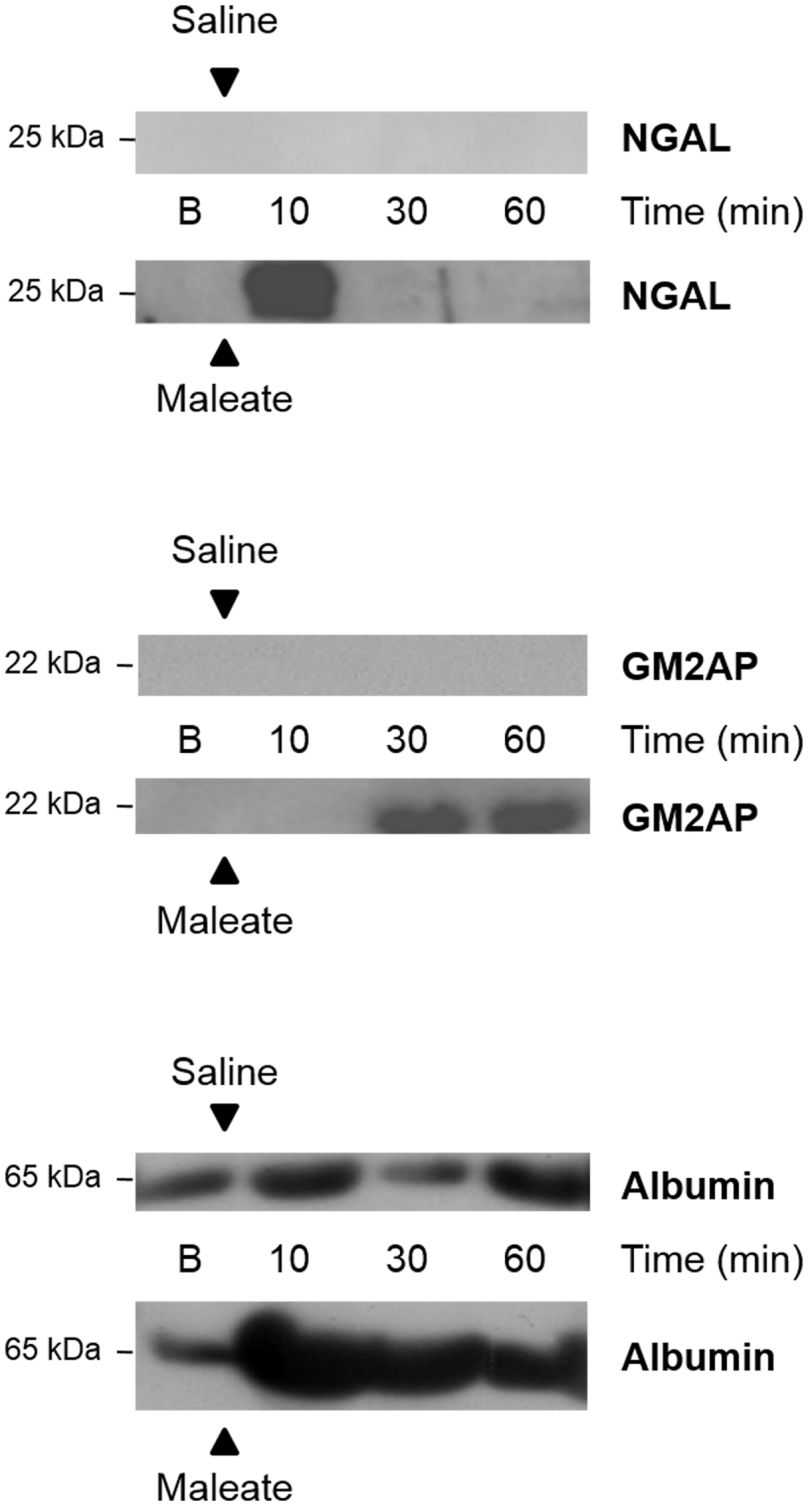
Effect of a single intravenous bolus of maleate (or saline as control) in the urinary excretion of NGAL, GM2AP and albumin during the following hour in normotensive-normoglycemic (Wistar) rats (n = 3 per group).

A potential cause of increased urinary excretion of NGAL would be its increased concentration in the blood, which we examined by western blot. We found no increment in NGAL plasma concentration in hyperglycemic SHR that could explain its increased urinary excretion (figure 6-C). Because low molecular weight proteins (such as NGAL) freely filtrate through the glomerular filtration barrier, the enhanced urinary excretion must be the consequence of altered tubular handling (i.e. reabsorption). *In vivo* inhibition of proximal tubule, megalin/cubilin-driven endocytosis with maleate produced only a brief and transient increase in urinary NGAL, while causing a more sustained excretion of albumin and ganglioside M2 activator protein (figure 8). This indicates that although the megalin/cubilin complex reabsorbs NGAL, other re-uptake mechanisms exist that redundantly participate in NGAL reabsorption.

## Discussion

Diabetes and hypertension frequently coexist, and their combination provides additive risk of chronic nephropathy, cardiovascular events and death [37]–[39]. As an example, diabetes is responsible for 36.8% of diagnosed cases of chronic kidney disease (CKD) in the USA [40]. It is thought that up to 15–30% of type 1 diabetic patients and 20–40% of type 2 will develop renal problems in the evolutionary course of diabetes [1]. Diabetes is the leading cause of ESRD; near 50% of ESRD patients are diabetic. Hypertension is the second leading cause of ESRD. About 51–63% of all patients with CKD are hypertensive. This number grows to 90% in patients over 65 years. In the corresponding general population the incidence of hypertension is 11–13% and 50%, respectively [1]. In this article, we propose uNGAL as a prospective marker of the additive risk of CKD posed by the combination of hypertension and diabetes.

NGAL is a 25 kDa protein of the lipocalin superfamily. This superfamily comprises proteins formed by 8 β-strands composing a β-barrel and enclosing a calyx, which binds and transports low-molecular-weight molecules [41]. In turn, NGAL binds and is transported by cell membrane transporters, such as the megalin-cubilin complex and 24p3 receptor [42]. It is expressed by different epithelia (including renal tubuli) in physiological and pathological circumstances [16], [43]. A relation has been proposed between inflammation and NGAL expression in neutrophils and epithelial cells [44]. In agreement, NGAL has been shown to be involved in the repair of ischemic renal tubular epithelium [36]. Indeed, treatment with exogenous NGAL ameliorates the kidney injury caused by ischemia-reperfusion [45]. This effect is thought to be mediated, at least in part, by favouring epithelial cell dedifferentiation, proliferation and, thus, repair. Strikingly though, NGAL-deficient mice are significantly protected against the chronic renal damage induced by 75% nephrectomy [46]. Interestingly, NGAL over-expression in these mice was mediated by hypoxia-inducible factor 1α (HIF-1α). It can be hypothesized that NGAL is expressed as a mediator of an inflammatory response, initially unleashed as a repair response. In such case, NGAL might act as a repair mediator. However, a persistent inflammatory response has been shown to be detrimental for the acute and chronic kidney repair (reviewed in 1 and 2). In those circumstances, NGAL might turn prejudicial overall. Moreover, NGAL has been proposed as a real-time indicator of the progression of chronic renal damage. NGAL also plays a role in the pathogenesis and clinical manifestations of atherosclerosis, acute myocardial infarction and heart failure. It has also been proposed as a potential link between the kidney and the cardiovascular system. In fact, cardiac, vascular and serum levels increase in a number of cardiovascular diseases [47], including those resulting from CKD.

Increasing serum and urinary NGAL correlate with decreasing glomerular filtration and with increasing renal parenchymal degeneration [26], [48]. Under these circumstances, the accumulation of NGAL in the blood is related to the reduced filtration; and its increase in the urine is thought to be the consequence of its increased expression by damaged renal compartments, mostly the tubuli. However, the origin of increased uNGAL in our model is not the renal parenchyma, which is not damaged by the time NGAL is detected in the urine, nor increased gene expression or protein levels are detected in renal tissue homogenates from hypertensive and hyperglycaemic animals (figure 6). When the blood is substituted by a protein-free isotonic solution (Krebs), no NGAL is detected in the urine of these animals. Furthermore, when NGAL is added to the Krebs solution in *in situ* perfusion experiments, hypertensive-hyperglycemic kidneys excrete more NGAL in the urine than those in hypertensive rats. This indicates that uNGAL comes from the blood and that there is an intrinsic alteration in the tubular handling of this protein in hypertensive-hyperglycemic rats, most probably a defect in its tubular re-uptake.

Because of its small size, NGAL filters freely through the glomerular barrier. Under normal conditions, filtered NGAL is reabsorbed in the tubules with the concourse of the proximal tubule endocytic complex formed by megalin and cubilin [49]. In this sense, our experiments show that, although the megalin/cubilin complex participates in NGAL reabsorption, there are other redundant mechanisms capable of achieving full NGAL reuptake shortly after megalin/cubilin voidance, as demonstrated in figure 7. As such, the key alterations caused by sustained hypertension and hyperglycemia leading to NGAL urinary over-excretion must be looked for in tubular handling systems different from that of megalin/cubilin. This differs from the mechanism of microalbuminuria in early diabetic nephropathy, which is mainly due to reduced reabsorption through the megalin system. Indeed, both reduced megalin expression [50], and disruption of megalin-dependent reuptake [51] are thought to mediate microalbuminuria, although the precise mechanism is not fully understood. On the contrary, the mechanism underlying hypertensive microalbuminuria is mostly unknown.

Our results point at a potentially new and different utility of NGAL as a diagnostic (or even prognostic) marker in the course of CKD. NGAL appears in the urine upon the chronically synergistic action of hypertension and hyperglycemia, even in the absence of overt nephropathy. In our experimental model, this capability is not achieved by albuminuria, which more closely correlates with hyperglycemia and increased urine output than with the additive presence of both risk factors (namely hypertension and hyperglycemia). Both NGAL and albumin are increasingly excreted with the urine as a result of incipient and subtle alterations in their renal handling. However, NGAL might incorporate a new capacity to early detect and quantify the increased risk posed by the synergistic action of hypertension and diabetes; and, prospectively, also to monitor the evolution and prognosis of CKD patients.

Clinical studies are necessary to ascertain the capacity of uNGAL to stratify patients according to their prognosis and their individual risk of progression through CKD, in early stages where no other signs different from microalbuminuria are evident. In perspective, this pre-emptive and personalized stratification would enable us to transform qualitative risk factors (same status for all hypertensive-diabetic patients) into quantitative parameters that score how hypertension and diabetes affect patients in an individual basis with respect to their specific prognosis on CKD progression.
